# Measuring Subcounty Differences in Population Health Using Hospital and Census-Derived Data Sets: The Missouri ZIP Health Rankings Project

**DOI:** 10.1097/PHH.0000000000000578

**Published:** 2018

**Authors:** Elna Nagasako, Brian Waterman, Mathew Reidhead, Min Lian, Sarah Gehlert

**Affiliations:** Division of General Medical Sciences, Washington University School of Medicine in St. Louis, St. Louis, Missouri (Drs Nagasako and Lian); Center for Clinical Excellence, BJC HealthCare, St. Louis, Missouri (Dr Nagasako); Missouri Hospital Association, Jefferson City, Missouri (Messrs Waterman and Reidhead); George Warren Brown School of Social Work, Washington University in St. Louis, St. Louis, Missouri (Dr Gehlert); and Alvin J. Siteman Cancer Center, St. Louis, Missouri (Drs Lian and Gehlert)

**Keywords:** health factors, health outcomes, health rankings, population health, public health surveillance, small-area health estimates, subcounty-level health estimates

## Abstract

**Context:**

Measures of population health at the subcounty level are needed to identify areas for focused interventions and to support local health improvement activities.

**Objective:**

To extend the County Health Rankings population health measurement model to the ZIP code level using widely available hospital and census-derived data sources.

**Design:**

Retrospective administrative data study.

**Setting:**

Missouri.

**Population:**

Missouri FY 2012–2014 hospital inpatient, outpatient, and emergency department discharge encounters (N = 36 176 377) and 2015 Nielsen data.

**Main Outcome Measures:**

ZIP code–level health factors and health outcomes indices.

**Results:**

Statistically significant measures of association were observed between the ZIP code–level population health indices and published County Health Rankings indices. Variation within counties was observed in both urban and rural areas. Substantial variation of the derived measures was observed at the ZIP code level with 20 (17.4%) Missouri counties having ZIP codes in both the top and bottom quintiles of health factors and health outcomes. Thirty of the 46 (65.2%) counties in the top 2 county quintiles had ZIP codes in the bottom 2 quintiles.

**Conclusions:**

This proof-of-concept analysis suggests that readily available hospital and census-derived data can be used to create measures of population health at the subcounty level. These widely available data sources could be used to identify areas of potential need within counties, engage community stakeholders, and target interventions.

Effectively engaging communities to address the social, economic, environmental, clinical, and behavioral factors that affect health is critical for improving population health outcomes. Assessments have identified subcounty neighborhood-scale measures of health and related factors as a data need.^1–4^ Multiple metrics, indices, and rankings have been developed for assessing community health, including community and neighborhood indicators, well-being indices, deprivation indices, and health indicators and rankings.^5–9^ However, few of these are widely available at the subcounty level.

Geographic variation in health outcomes and related risk factors at the small-area level (eg, substate and subcounty) has received increasing attention.^10–15^ Local-level primary data collection and aggregation are important for obtaining small-area data.^1,6,16,17^ Alternatively, extension of county-level population measures to the subcounty level using small-area estimation techniques can address this need.^15,18–23^ However, these approaches can be costly, resource intensive, or impeded by sampling constraints. This exploratory proof of concept study was funded by County Health Rankings (CHR) to (1) design a method to extend the CHR population health measurement framework to the ZIP code level using widely available data; (2) examine the agreement between published CHR indices and ZIP-derived measures reapportioned to the county level; and (3) quantify the subcounty variation at the ZIP code level in Missouri.

## Materials and Methods

### Data sources

The primary study aim was to evaluate whether hospital and census-based data sets could be used within the CHR framework to create health factors and health outcomes indices at the ZIP code level in Missouri. Candidate model input variables were gathered from Missouri Hospital Association, Hospital Industry Data Institute FY 2012-FY 2014 (October 1, 2011 to September 30, 2014) hospital inpatient, outpatient, and emergency department discharge databases for Missouri residents (N = 36 176 377) and linked by reported residential ZIP code to Nielsen sociodemographic data that use spatially defined census block group-to-ZIP code correspondence.^24^ Administrative hospital discharge data are commonly used in public health applications such as disease surveillance programs,^25–33^ feature standardized record layouts, and are widely available.^34^ Candidate variables for the CHR socioeconomic domain were gathered primarily from the 2015 Nielsen Pop-Facts Premier database,^24^ which provides intercensal estimates based on block group-level American Community Survey data. The socioeconomic health factor domain was augmented with a socioeconomic deprivation index developed by Schootman, Lian, and colleagues.^35–37^ Supplemental Digital Content Table 1, available at http://links.lww.com/JPHMP/A317, displays all candidate and retained model variables, external validation variables, and data sources by CHR domain and subdomain.

Employment of the aggregate data utilized as model inputs was governed by Hospital Industry Data Institute master data use agreements. Academic personnel participation was reviewed by the Washington University School of Medicine Human Research Protection Office.

### Measures

#### Conceptual framework

Variables were extracted on the basis of correspondence to the CHR conceptual framework,^38^ which includes a health factors domain with 4 subdomains (health behaviors, clinical care, social and economic factors, and physical environment) and a health outcomes domain with 2 subdomains (length of life and quality of life). *International Classification of Diseases, Ninth Revision Clinical Modification* codes for variables drawn from hospital data were identified through literature review, keyword search within diagnosis code descriptions, and expert input. A detailed description of variables evaluated for each CHR domain is included in the Supplemental Digital Content Table 1, available at http://links.lww.com/JPHMP/A317.

### Data analysis

#### Evaluation of candidate model inputs

Descriptive statistics and pairwise correlations were conducted to initially evaluate standardized candidate model inputs from hospital and Nielsen data sources against county-level CHR indices. Candidate measures with highly skewed distributions and nonpositive pairwise correlations were further evaluated and transformed or eliminated. Pairwise correlations with external validation measures and CHR county-level subdomain scores were used to further reduce candidate measures sets; only measures with statistically significant pairwise correlations of 0.20 or greater with assigned CHR subdomain scores were retained. Injury-related mortality was retained despite a low correlation to ensure conceptual domain coverage in principal components analyses. Table 1 contains a list of the candidate variables that were retained for inclusion in our final model.

#### Model creation and evaluation at the county level

Principal components analysis was applied to subdomain input sets to derive subdomain analog factor scores.^39,40^ Each yielded only 1 principal component with an eigenvalue greater than 1, indicating that a component was sufficient to explain the common variation in subdomain indicator groups. Linear regression was then used to model CHR Health Factors and Health Outcomes scores as a function of derived subdomain analog factor scores. Consistent with the CHR conceptual framework, the Health Factors model included derived factor scores for Behavior, Environment, Clinical Access, and Socioeconomic Status subdomains as independent variables. The Health Outcomes model included scores from the Quality of Life and Mortality subdomains supplemented with predicted scores from the regression model for Health Factors, for improved model fit, and predictive accuracy. Predicted values from the 2 regression models served as derived county-level analog scores for CHR Health Factors and Health Outcomes. Pairwise Pearson correlations and scatterplots were used to assess strength of association between analog scores and CHR scores.

#### Model creation and evaluation at the ZIP code level

Principal components analysis was applied to each ZIP-level input data set to derive ZIP-level CHR subdomain analog scores, using an identical approach to that used for the county-level analysis. Each ZIP-level subdomain model yielded a single component score with an eigenvalue greater than 1. Summarization of ZIP-level estimates into county-level estimates allowed comparison of ZIP-derived results with the CHR results. This spatial interpolation was facilitated using a ZIP (“source” zone) to county (“target” zone) weighting file derived by allocating ZCTA (the census surrogate of ZIP codes based on whole census blocks) population totals to counties based on ZCTA-county proportional allocations using the MABLE/Geocorr version 12 engine.^41^ The process was applied to all Missouri ZIP codes and provided an empirical basis for handling codes that overlap counties.

ZIP-level Health Factors and Health Outcomes domain scores were computed using regression weights derived from county-level analyses. General linear mixed modeling was used to model derived ZIP-level CHR Health Factors and Health Outcomes scores as a function of random county-level intercepts. ZIP-to-County mapping weights produced intraclass correlation estimates of the variation in ZIP-level scores explained by within-county clustering. These weights produced Best Linear Unbiased Predictors of county-level Health Factors and Health Outcomes scores.

Pairwise Pearson correlation coefficients and scatterplots were used to assess the strength of correlation between ZIP-derived analog scores reapportioned to the county-level and corresponding CHR scores. Cross-classification tables, weighted *κ* statistics, and agreement plots were used to assess pairwise agreement between quintile-ranked CHR scores and corresponding derived analogs. Mapped displays of CHR and ZIP-derived quintile-ranked results were produced for visual comparison.

#### Assessment of intracounty variation at the ZIP code level

The proportion of variation at subcounty level was assessed to determine within-county variation using model-based intraclass correlations. Concordance between the ZIP code and county-level quintile ranking in health factors and health outcomes was also assessed.

### Advisory group

This project was informed by an advisory group comprising local public health, philanthropic, hospital association, hospital community benefit, academic, and community advocate organization members. Quarterly meetings were held to obtain ongoing immediate feedback on this project’s aims, methods, and results to ensure that the study’s approach would address the differing needs of multiple stakeholders.

## Results

### Evaluation of candidate model inputs

We found that model inputs drawn from hospital and census-derived data were significantly correlated with CHR indices. Table 1 contains summary statistics and correlation with CHR subdomains for all candidate variables retained as final model inputs. All pairwise correlations were statistically significant at *P* < .05, with the exception of injury-related mortality, which was retained to help ensure Environment subdomain coverage.

### Model creation and evaluation at the county level

Principal components analysis produced models for health factors and health outcomes using hospital and commercial data with moderate to substantial correlations with CHR domain and subdomain scores, yielding 1 significant factor for each subdomain. Variable inputs, principal component eigenvalues, and pairwise correlations with CHR subdomain scores for 114 Missouri counties are displayed in Supplemental Digital Content Table 2, available at http://links.lww.com/JPHMP/A317. One Missouri county lacked sufficient data and was excluded. Correlations between CHR Health Outcomes and Health Factors overall domain scores and county-level indices calculated from derived subdomain analog scores using standard CHR weights are displayed in Table 2. All pairwise correlations were statistically significant (*P* < .05).

Quintile agreement between CHR Health Factors and the derived analogs for 114 Missouri counties was 54%, with 92% of counties landing within 1 quintile (weighted *κ* = 0.66, *P* < .05). For Health Outcomes, 43% of counties fell within the same quintile and 89% of ZIP-derived analogs fell within 1 quintile difference of the CHR results for 2015 (weighted *κ* = 0.54, *P* < .05). Similar agreement was observed when evaluated as octiles and deciles. Scatterplots and correlations between CHR Health Outcomes and Health Factors overall domain scores and county-level indices calculated from derived subdomain analog scores using standard CHR weights are displayed in Supplemental Digital Content Figures 1 and 2, available at http://links.lww.com/JPHMP/A317.

### Model creation and evaluation at the ZIP code level

Concordance between ZIP code–level health factors and health outcomes scores for Missouri derived from hospital and commercial data sets and the original CHR county scores was evaluated by interpolating the ZIP code results to the county level. We found moderate to substantial, statistically significant agreement between the published CHR indices and the derived indices. Correlations between CHR Health Outcomes and Health Factors overall domain scores and ZIP-level analogs calculated from derived subdomain analog scores using standard CHR weights are displayed in Table 2. All pairwise correlations were statistically significant (*P* < .05).

**FIGURE 1:**
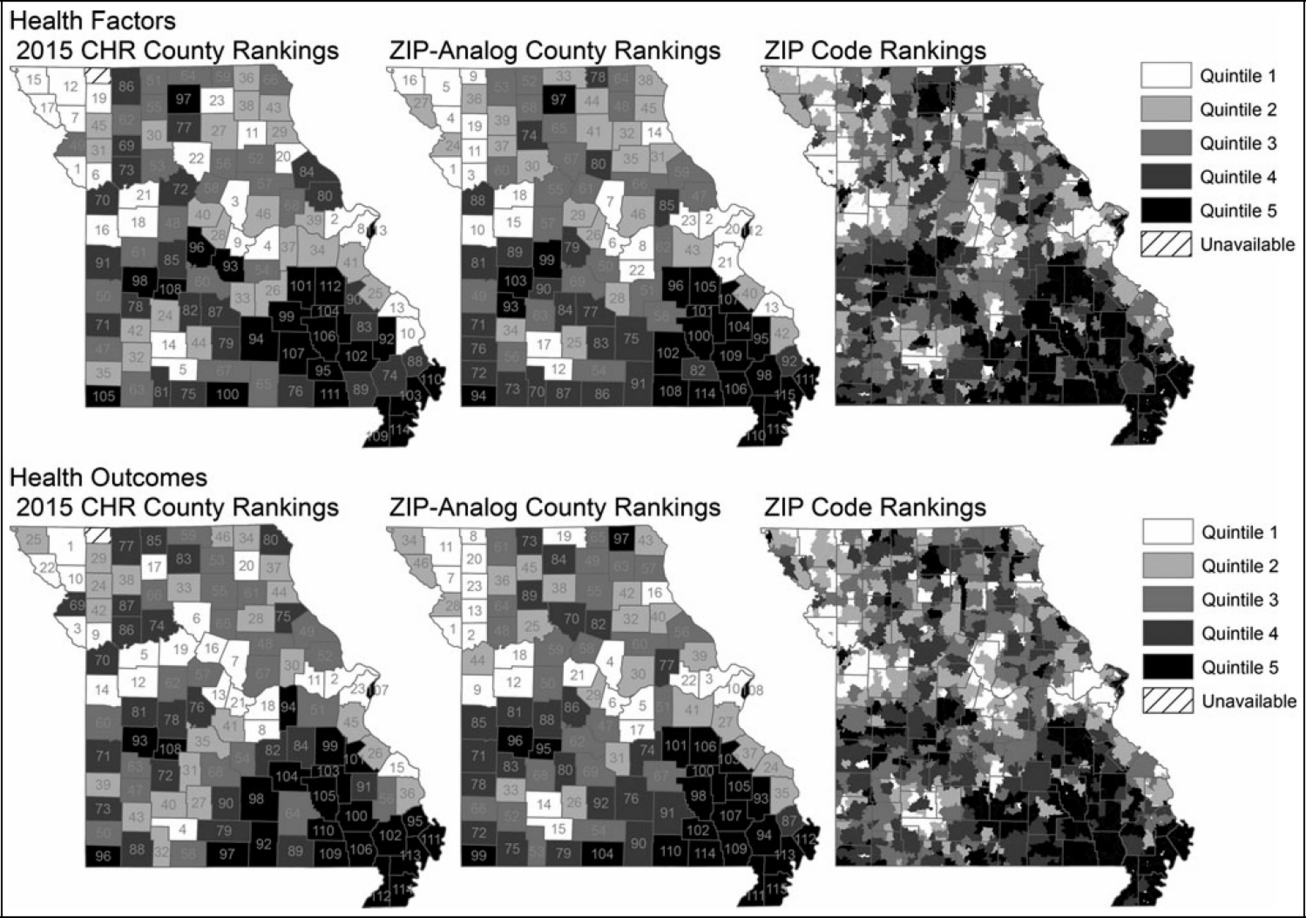
County and ZIP-level rankings derived from hospital and census-derived data sets vs. 2015 county health rankings results

Quintile agreement between CHR Health Factors and the ZIP-derived analogs for Missouri counties was 52% with 94% of counties landing within 1 quintile (weighted *κ* = 0.66, *P* < .05) (see Supplemental Digital Content Figure 3, available at http://links.lww.com/JPHMP/A317). For Health Outcomes, 49% of counties fell within the same quintile and 84% of ZIP-derived analogs fell within 1 quintile of the 2015 CHR results (weighted *κ* = 0.56, *P* < .05) (see Supplemental Digital Content Figure 4, available at http://links.lww.com/JPHMP/A317). Mapped representations of ZIP-level ranking quintiles for Missouri based on derived Health Factors and Health Outcomes indices versus 2015 CHR indices are displayed in Figure 1 (color version available in Supplemental Digital Content Figure 5, available at http://links.lww.com/JPHMP/A317).

### Evaluation of subcounty variation in health factors and outcomes

**FIGURE 2:**
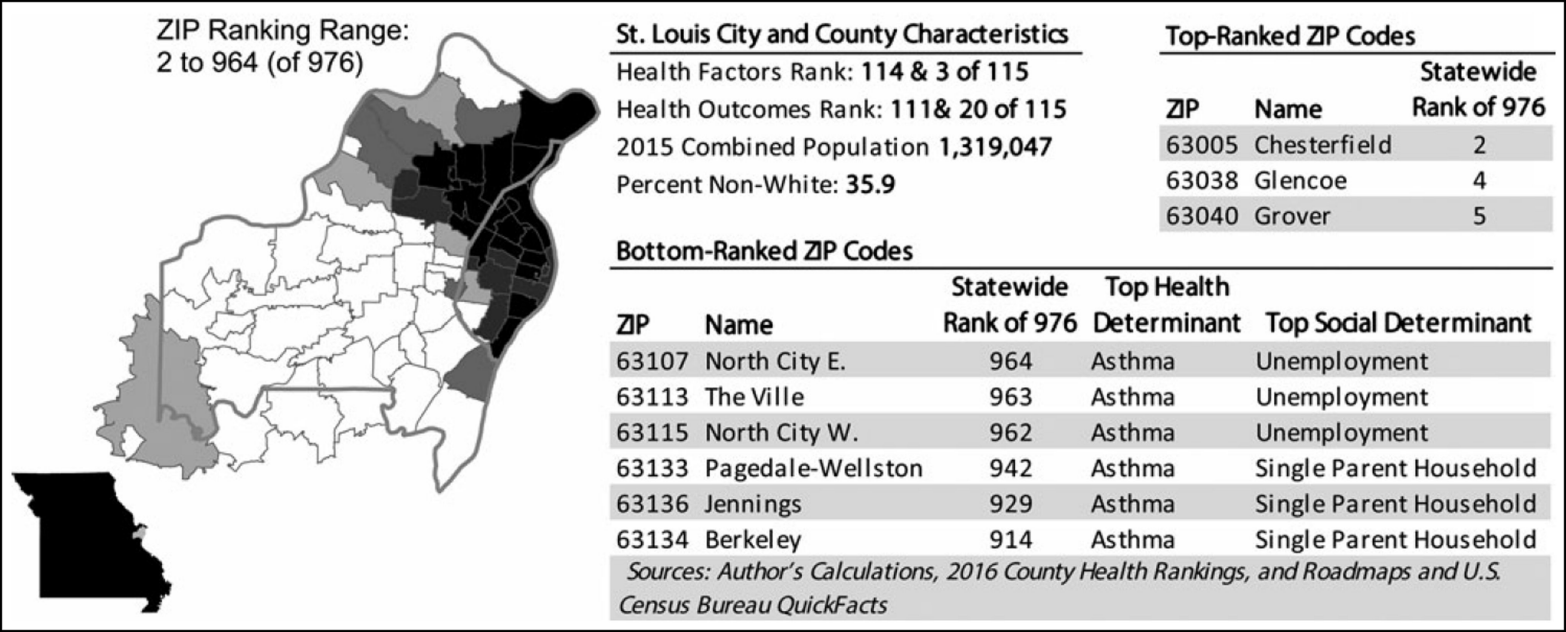
Subcounty variation in health factors and outcomes in urban St. Louis City & County, Missouri

**FIGURE 3:**
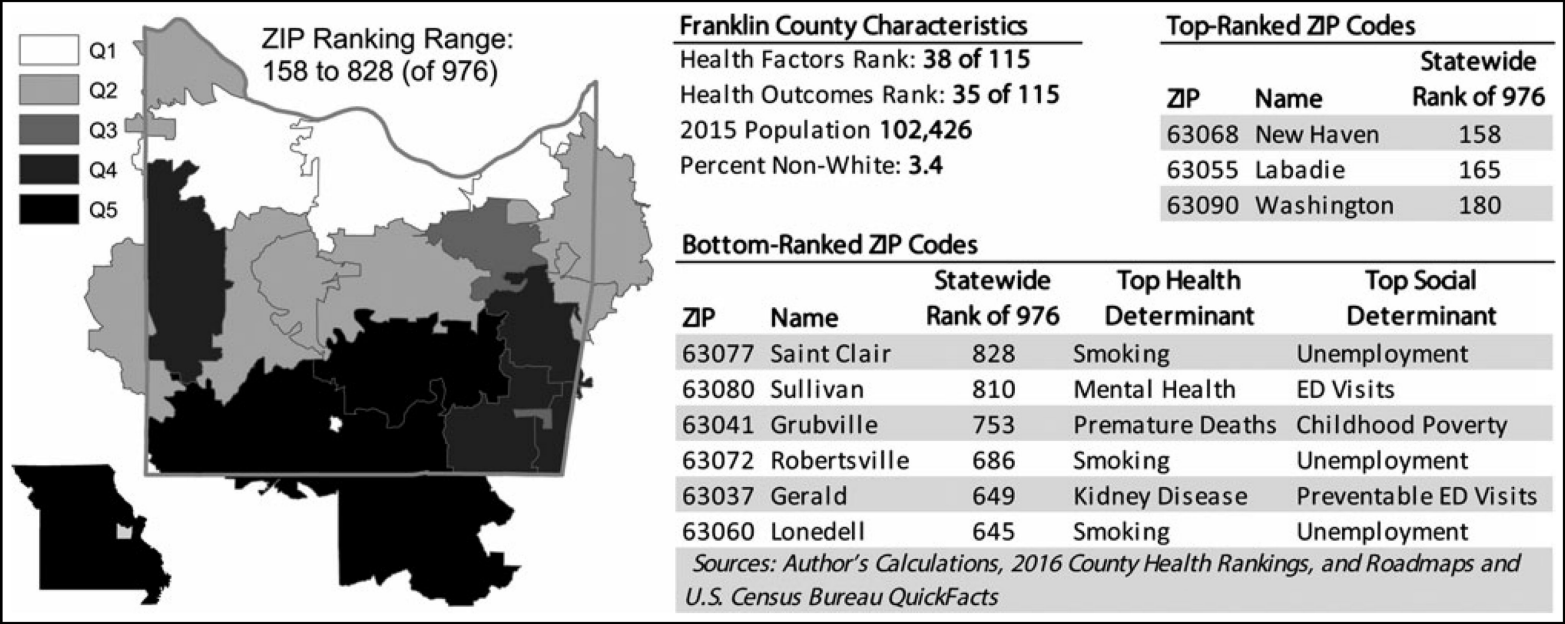
Subcounty variation in health factors and outcomes in rural Franklin County, Missouri

ZIP code–level health factors and outcomes indices showed substantial variation at the ZIP code level. Mixed models yielded intraclass correlation estimates associated with county-level random intercepts of 0.44 and 0.5 for Health Factors and Health Outcomes, respectively, indicating that 50% to 56% of the variance in derived domain scores is observed at the ZIP code level. Substantial variation was observed at the ZIP code level, with 20 (17.4%) counties having ZIP codes in both the top and bottom quintiles of health factors and health outcomes. Thirty of the 46 (65.2%) counties in the top 2 quintiles had ZIP codes in the bottom 2 quintiles. This was observed in both urban and rural areas (see Figures 2 and 3; color versions available in Supplemental Digital Content Figures 6 and 7, available at http://links.lww.com/JPHMP/A317).

## Discussion

Using the conceptual framework of the County Health Rankings & Roadmaps,^38^ we identified candidate measures available at the ZIP code level in hospital discharge and commercial census-derived data sets for each CHR health factors and health outcomes domain and subdomain. We derived ZIP code–level health factors and health outcomes scores for Missouri using these measures. We evaluated concordance with the original CHR indices at the county level by apportioning the ZIP code results to the county level. Finally, we assessed the extent of subcounty variation in health factors and outcomes indicated by these indices. The results of this exploratory study show statistically significant agreement between published CHR indices and ZIP code–level indices derived using hospital and census-derived data that are widely available at subcounty levels. Although the degree of agreement was limited, this finding suggests that despite the limitations of hospital data in capturing population health, these data can be combined with other sources to assess variation in health at the subcounty level. These findings serve as a starting point for further work to develop data sources for use by community stakeholders working to improve health in communities. In addition, mixed-models results and graphical displays illustrate how these health factors and health outcomes indices vary at subcounty levels (see Figures 1–3; color versions available in Supplemental Digital Content Figures 5–7, available at http://links.lww.com/JPHMP/A317). This underscores the potential for subcounty data to identify small-area variation and target scarce community health improvement resources.

It is important to note that the results of this exploratory study are not presented to suggest the model and measures we derived are replications of or alternatives to CHR constructs. Rather, they are offered as “proof-of-concept” evidence that selected data sources can be used in the context of the CHR framework to derive alternative measures that correspond sufficiently with established county-level rankings to support their plausible use as a basis for assessing subcounty variation demonstrably associated with CHR domains. Although hospital data will not completely capture population health, local decision making around public health of necessity often involves hospitals and hospital data. Given the origin of our approach in the needs of hospital-public health partnerships, the objective of our exploratory study was to apply these data in a way that was appropriate and realistic for local stakeholder groups. We assert that the pattern and magnitude of presented correlations between CHR and our derived analog measures support further exploration of the practical use of these measures as a basis for identifying subcounty areas of higher and lower relative community health need.

Geographic variation in health factors and outcomes at the small-area level, including the substate, subcounty, and census tract levels, has been noted in many contexts.^10–12^ Subcounty health data and their drivers can be used by local public health departments to inform policy decisions and engage community partners. Yet, data are often unavailable at the ZIP code or census tract levels, and small area data have been identified as a need at both the local and federal levels.^1–4,6^ Primary data collection using surveys at the subcounty level and the application of small-area estimation techniques to existing survey results are an important source of subcounty data.^1,16,17^ However, data collection can be costly and resource intensive^1^ and small-area estimation may be impeded by the lack of rural data, the potential discordance between geographic areas and the area boundaries of interest, and the area-level context effect on outcomes independent of individual characteristics.^17^

Hospital administrative data have been used for public health surveillance and the detection of geographic variation in factors and outcomes.^11,12,25–33^ An assessment of chronic disease prevalence using emergency department administrative data found rates comparable with those from survey data and that significant neighborhood variation in diabetes burden was identifiable using this method.^12^ Given the importance of sociodemographic determinants,^9^ we added Nielsen data to hospital administrative data. Our resulting ZIP code–level indices for the CHR domains and subdomains in Missouri agreed with our predefined gold standard of the published CHR county-level measures and identified significant subcounty-level variation. This supports the continued exploration of hospital administrative data as a subcounty-level source and identifies a method for combining hospital and commercial data sets to produce factors and rankings scalable to other states. Geographically based health rankings and indicators have been used to draw media attention to public health issues, including health disparities, and to engage communities in partnerships to improve health,^7,8,42,43^ suggesting that ZIP code–level measures can be used for engagement of communities to address identified subcounty data needs.^1,4^ In addition, there is a need for the alignment of hospital community benefits spending with community needs.^44–47^ Use of data sets readily available to hospitals to produce public health data can help support these alignment efforts via the engagement of hospital stakeholders.

## Limitations

Our approach has limitations. First, because a goal was to use data readily accessible in other states, we did not use data sources arguably more directly related to the CHR domains, yet not widely available across states. For example, the restriction to hospital and census-derived data limited the measures available in the environmental health factors subdomain, which has been identified as a challenge for the CHR rankings themselves.^48^ Future iterations of these models will investigate the incorporation of additional variables in the environmental domain such as particulate matter, land use, and land cover data. Second, the use of hospital administrative data in public health settings has limitations.^27,30,32,33,49,50^ The population pool for hospital data sets may not be as representative of the general population as population-based surveys. Although rural data availability is a hospital data strength, low population sometimes meant lack of ZIP code–level data. Also, in both rural and nonrural settings, patients with financial, spatial, and access barriers may seek hospital care at increased or decreased rates, yielding selection bias. The majority of hospital emergency department Medicaid and Medicare patient visits are considered nonurgent—with a diagnosis of acute upper respiratory infection most common. This may present autocorrelation between our measures of hospital utilization and socioeconomic factors. Third, although address is collected as a routine part of billing, patient ZIP code was sometimes unavailable. Some missing ZIP code data may be attributable to errors in data collection; the data generation process also excludes patients who are homeless or decline to provide information on residence. However, this occurred in a relatively small proportion of our discharge records (0.08%) and is unlikely to play a major role in our analysis. In addition, Nielsen data are estimated with American Community Survey block group-level estimates and subject to similar sampling bias. While Nielsen uses enhanced aggregation and distribution techniques to minimize this bias, the potential for artificial spreading of actual variance may arise as a result. Nielsen data are readily available to hospitals as they are widely used for strategic planning purposes, and the use of Nielsen databases can reduce time spent on data extraction; however, the cost of these data could be prohibitive to health departments or other organizations that do not already have access to these data. We anticipate that the substitution of Nielsen data with publicly available American Community Survey data would yield similar results; this is a topic for further investigation. Also, only a single data set encompassing 1 state was used in the study. Finally, our approach is subject to the limitations inherent in producing health rankings for community engagement, including the proliferation of measures encountered by local communities and the need to link the data to community action by ensuring its meaningfulness.^2,5,6,51^

## Conclusion

We demonstrated that hospital and census-derived data can be used to extend a commonly used framework for county-level population health to subcounty areas. Although hospital data reflect only a subset of the health of a community, this study suggests that its use in combination with other sources warrants further exploration as a subcounty data source. Our future work will include making these results publicly available using an interactive platform, evaluating their use in meeting data needs of Missouri stakeholders, and evaluating performance of this method on successive years of data, in other states, and with the inclusion of additional data sources for domains not readily captured in hospital and census-derived data sets.

## Supplementary Material

Supp. File 1

## Figures and Tables

**TABLE 1:** Summary Statistics and Correlation With CHR Subdomains for Hospital and Census-Derived Data Set Measures Retained as Final Model Inputs^a^

Domain	Subdomain	Measure	Mean (SD)	CHR Correlation
Health outcomes	Mortality	Premature death	5.7 (1.5)	0.66
		Years productive life lost	101.2 (30.2)	0.69
	Quality of Life	ED visits	1371 (356.7)	0.34
		IP visits	416.4 (74.7)	0.42
		Low birth weight	55.1 (16.3)	0.21
		Psychiatric diagnoses	30.6 (11.2)	0.45

Health factors	Behavior	Teen pregnancy	35 (13.1)	0.63
		Sexually transmitted infections	57.7 (24.5)	0.27
	Clinical Access	Off-hours ED visits	0.4 (0.1)	0.33
		Health care worker density	23.7 (7.1)	0.51
		AHRQ PQI total	49.8 (15.6)	0.56
	Environment	Assault diagnoses	12.0 (5.8)	0.39
		Injury-related mortality	2.6 (0.7)	0.12
	Socioeconomic Status	Education less than high school	0.16 (0.1)	0.68
		Unemployment	0.09 (0.03)	0.62
		Childhood poverty rate	0.21 (0.07)	0.71
		Median HH income	42 667 (7995)	0.69
		Socioeconomic deprivation	0 (1.1)	0.72

Abbreviations: AHRQ, Agency for Healthcare Research and Quality; CHR, County Health Rankings; ED, emergency department; HH, household; IP, inpatient; PQI, Prevention Quality Indicators.

a One Missouri county was excluded because of insufficient data.

**TABLE 2:** Correlation Between CHR Domain Scores and Domain Scores Derived From Hospital and Census-Derived Data Sets for 114 Missouri Counties^a^

	CHR Health Factors	CHR Health Outcomes
Model results derived from county-level data		
Health Factors Analog	0.88	0.79
Health Outcomes Analog	0.85	0.83

Model results derived from ZIP code–level data^b^		
Health Factors Analog	0.87	0.82
Health Outcomes Analog	0.84	0.83

Abbreviation: CHR, County Health Rankings.

a One Missouri county was excluded due to insufficient data.

b ZIP code–level model results proportionally allocated and summarized to county level.

## Implications for Policy & Practice

■ The use of widely available hospital discharge data supplemented with census-derived area-level data could allow potential application of this method of producing subcounty data in other states.
■ Estimates of health factors and outcomes at the ZIP code level can target resources to areas most in need and engage community members using more geographically focused data.
■ If applied in an ongoing way, this approach could facilitate evaluations of the effectiveness of population health improvement efforts at the subcounty level.
