# Supplementary material for: Measuring Subcounty Differences in Population Health Using Hospital and Census-Derived Data Sets: The Missouri ZIP Health Rankings Project
Source: J Public Health Manag Pract. Author manuscript; Available in PMC 2018 Jul 1. (PMC5704978; doi:10.1097/PHH.0000000000000578)
Supplement: Supp. File 1 [file NIHMS892743-supplement-Supp__File_1.pdf]

**Supplemental Digital Content: Measuring sub-county differences in population health using hospital and census-derived data sets: the Missouri ZIP Health Rankings project**

**Description of ZIP Code-Level Candidate Model Variables Evaluated by County Health Rankings Domain:**

*Health Outcomes:* The candidate variable data set for the health outcomes domain was drawn from hospital discharge data. Mortality subdomain variables were based on years of potential life lost and number of hospital deaths prior to age 75, measured as a rate of the population. In-hospital deaths were identified using discharge codes indicating expiration during the hospitalization. Quality of life subdomain variables included 1) overall rates of hospital utilization; 2) indicators of neonatal health based on ICD 9-CM codes diagnosing low birth weight, preterm births, light body weight for age, infant malnutrition, delayed growth and immaturity; 3) rates of hospital utilization for mental health-related diseases and disorders as indicated by Major Diagnostic Category (MDC) 19; and 4) rates of hospital diagnoses for eleven chronic conditions based on the Agency for Healthcare Research and Quality's Clinical Classification Software (AHRQ CCS) using definitions from the Missouri Department of Health and Senior Services (DHSS).

*Health Factors:* Candidate variables were categorized into four subdomains in accordance with the CHR population health framework.[29] The health behaviors subdomain included population-based rates of hospital diagnoses for primary and secondary tobacco smoke exposure, obesity, alcohol and substance abuse (MDC 20), sexually transmitted infections and teen births or pregnancies, as indicated by female patients under age 20 with a hospital encounter for pregnancy, childbirth and puerperium (MDC 14). Behavioral variables not identified with MDC codes were identified using arrays of ICD 9-CM codes.

The clinical care subdomain included candidate variables drawn from hospital discharge records and Nielsen, including the 1) rates of potentially avoidable emergency department utilization identified with the NYU ED Classification Algorithm[30]; 2) rate of hospital utilization by

uninsured patients as indicated by expected primary payer codes of self-pay or charity care; 3) rate of after-hours emergency department visits; 4) percent of the civilian population employed in health care; 5) rate of high risk mammography occurring in hospital settings; and 6) preventable hospitalizations identified with the AHRQ Prevention Quality Indicator methodology.

The social and economic factors subdomain was drawn primarily from Nielsen data. Education was measured with the percent of the population age 25 and older with less than a high school education, a high school education, or some college education. Candidate employment variables included the unemployment rate and percent of the labor force in a blue collar occupation. County- and ZIP-code-level income and poverty was measured with the poverty rate for families with children, income inequality as measured by the ratio of median household income for white and black families, overall median household income, median home values, and the rate of hospital utilization by Medicaid-eligible patients. The candidate social support variables included average household size and the percentage of female-headed households.

Candidate variables in hospital and Nielsen data for the environmental health factors were limited. For the health factor subdomain candidate variables for environmental conditions we used available data which included hospital utilization for asthma, the rate of injury-related mortality, the rate of assault-related hospital diagnoses, the housing vacancy rate and the percent of renter-occupied housing units.

**Supplemental Digital Content Table 1. Data Elements and Sources**

| Domain | | Measure | Source | Final Model | Purpose |
|---|---|---|---|---|---|
| Health Outcomes | Mortality | Rate of years of potential life lost (YPLL) before age 75 occurring in a hospital setting (ZIP and County-Level) | J | Y | Model Input |
| | | Rate of deaths before age 75 occurring in a hospital setting (ZIP and County-Level) | J | Y | Model Input |
| | | Rate of YPLL before age 75 (ZIP and County-Level) | M | | External Validity |
| | | Rate of YPLL before age 75 for external validation (County-Level) | G | | External Validity |
| | | Rate of YPLL before age 75 for external validation (ZIP-Level) | I | | External Validity |
| | QOL | Prevalence of hospital diagnosis of various chronic conditions (ZIP and County-Level) | J | | Model Input |
| | | Inpatient and ED Hospital utilization rates (ZIP and County-Level) | J | Y | Model Input |
| | | Prevalence of low birth-weight and high-risk pregnancies (ZIP and County-Level) | J | Y | Model Input |
| | | Hospital utilization rates for mental health disorders (ZIP and County-Level) | J | Y | Model Input |
| | | Prevalence of self-reported chronic conditions (County-Level) | H | | External Validity |
| | | Prevalence of self-reported physical health status (County-Level) | H | | External Validity |
| | | Prevalence of self-reported mental health status (County-Level) | H | | External Validity |
| | | Prevalence of low birthweight and preterm births (ZIP and County-Level) | F | | External Validity |
| Health Factors | Health Behaviors | Diagnosed smoking rate (ZIP and County-Level) | J | | Model Input |
| | | Diagnosed obesity rate (ZIP and County-Level) | J | | Model Input |
| | | Diagnosed alcohol/substance abuse rate (ZIP and County-Level) | J | | Model Input |
| | | Teen birth/pregnancy rate (ZIP and County-Level) | J | Y | Model Input |
| | | Diagnosed STI rate (ZIP and County-Level) | J | Y | Model Input |
| | | Adult smoking rate (County-Level) | H | | External Validity |
| | | Adult obesity rate (County-Level) | B | | External Validity |
| | | Excessive drinking rate (County-Level) | A | | External Validity |
| | | Teen birth rate (County-Level) | L | | External Validity |
| | | Rate of newly diagnosed chlamydia cases (County-Level) | N | | External Validity |
| | Clinical Care | Population 16+ employed in health care occupation (ZIP and County-Level) | O | Y | Model Input |
| | | Rate of uninsured hospital visits (ZIP and County-Level) | J | | Model Input |
| | | Rate of potentially avoidable ED visits using the NYU ED Classification Algorithm (ZIP and County-Level) | J | | Model Input |
| | | Rate of after hours ED visits (ZIP and County-Level) | J | Y | Model Input |
| | | Uninsured rate under age 65 (County-Level) | Q | | External Validity |
| | | HPSA and MUA/P Designation (County-Level) | P | | External Validity |
| | | AHRQ Prevention Quality Indicators (ZIP and County-Level) | K | Y | Model Input |
| | | Rate of Mammographies for High-Risk Patients (ZIP and County-Level) | J | | Model Input |
| | | Rate of preventable hospitalizations (County-Level) | D | | External Validity |
| | | Rate of female Medicare enrollees ages 67-69 that receive mammography | D | | External Validity |
| | Environment | Rate of diagnosed assault-gun, weapon, rape, etc. (ZIP and County-Level) | J | Y | Model Input |
| | | Rate of hospital mortality for injury (ZIP and County-Level) | J | Y | Model Input |
| | | Rate of hospital utilization for asthma (ZIP and County-Level) | J | | Model Input |
| | | Housing vacancy rate (ZIP and County-Level) | O | | Model Input |
| | | Renter occupied housing rate (ZIP and County-Level) | O | | Model Input |
| | Socioeconomic Status | Percent age 25+ with less than high school (ZIP and County-Level) | O | Y | Model Input |
| | | Unemployment rate (ZIP and County-Level) | O | Y | Model Input |
| | | Housing Characteristics (ZIP and County-Level) | O | | Model Input |
| | | Childhood poverty rate (ZIP and County-Level) | O | Y | Model Input |
| | | Median Household Income (ZIP and County-Level) | O | Y | Model Input |
| | | Rate of Medicaid hospital visits (ZIP and County-Level) | J | | Model Input |
| | | Household composition (ZIP and County-Level) | O | | Model Input |
| | | High school graduation Z-score (County-Level) | C | | External Validity |
| | | Some college (County-Level) | C | | External Validity |
| | | Unemployment Z-score (County-Level) | C | | External Validity |
| | | Severe housing problem Z-score (County-Level) | C | | External Validity |
| | | Children in poverty Z-score (County-Level) | C | | External Validity |
| | | Income inequality Z-score (County-Level) | C | | External Validity |
| | | Children in single-parent households Z-score (County-Level) | C | | External Validity |
| | | Violent crime Z-score (County-Level) | C | | External Validity |
| | | Injury deaths Z-score (County-Level) | C | | External Validity |
| | | Pre-validated small-area socioeconomic deprivation index (ZIP and County-Level) | E | Y | Model Input |

**Sources**

| | |
|---|---|
| A | BRFSS (2006 - 2012) |
| B | CDC Diabetes Interactive Atlas (2011) |
| C | County Health Rankings and Roadmaps (2015) |
| D | Dartmouth Atlas of Health Care (2012) |
| E | Author 2011 [details removed for peer review] |
| F | Missouri Department of Health and Senior Services, Birth MICA (multi-year through 2013) |
| G | Missouri Department of Health and Senior Services, Bureau of Vital Records (multi-year through 2012) |
| H | Missouri Department of Health and Senior Services, County-Level Study (2002-2003, 2007, 2011) |
| I | Missouri Department of Health and Senior Services, Death MICA (multi-year through 2013) |
| J | Missouri Hospital Association, HIDI Inpatient and Outpatient Hospital Discharge Databases (multi-year through 2014) |
| K | Missouri Hospital Association, HIDI Inpatient Hospital Discharge Databases (multi-year through 2014) |
| L | National Center for Health Statistics - Natality files (2006 - 2012) |
| M | National Center for Health Statistics (multi-year through 2013) |
| N | National Center for HIV/AIDS, Viral Hepatitis, STD, and TB Prevention (2012) |
| O | Nielsen-Claritas PopFacts Premier (multi-year through 2014) |
| P | U.S. Health Resource Services Administration (2015) |
| Q | US. Census SAHIE (2013) |

**Supplemental Digital Content Table 2. Pairwise Correlations Between Derived and CHR Subdomain Scores for 114 MO Counties**

| Subdomain | Key Input Variables | Input Variance Explained | CHR Subdomain Correlation |
|---|---|---|---|
| **Mortality** | Premature Death Rate, Years Productive Life Lost (YPLL) | 0.93 | 0.64 |
| **Quality of Life** | ED visits, IP Visits, Low Birthweight, Psychiatric Diagnoses | 0.59 | 0.5 |
| **Behavior** | Teen Pregnancy, Sexually-Transmitted Infections | 0.71 | 0.58 |
| **Clinical Access** | Off Hours ED visits, Healthcare worker density, AHRQ PQI Total | 0.49 | 0.62 |
| **Environment** | Assault Diagnoses, Injury-related Mortality | 0.59 | 0.34 |
| **SES** | Education < HS, Unemployment, Kids in Poverty, Median HHY, Socioeconomic Deprivation Index | 0.74 | 0.79 |

*One Missouri county was excluded due to insufficient data.

**Supplemental Digital Content Figure 1. Scatter Plot of 2015 CHR and ZIP Analog Derived Health Outcomes Z-Scores for Missouri Counties**

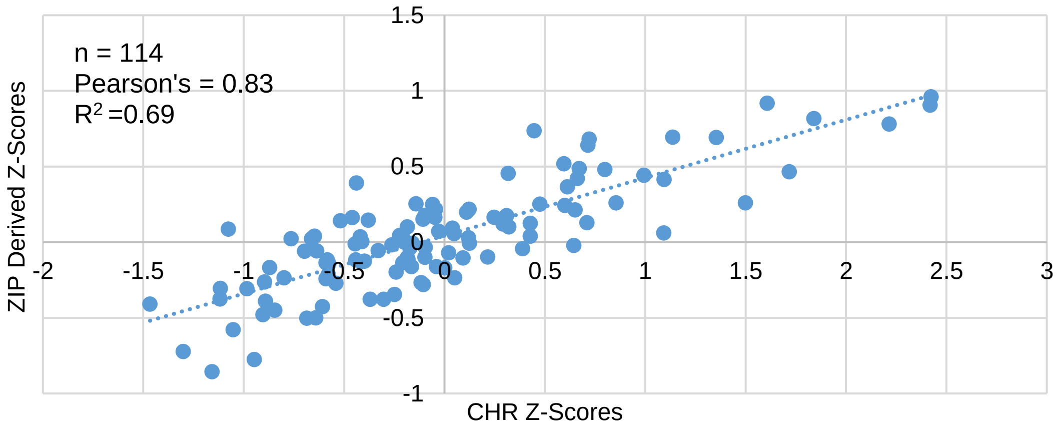


**Supplemental Digital Content Figure 2. Scatter Plot of 2015 CHR and ZIP Analog Derived Health Factors Z-Scores for Missouri Counties**

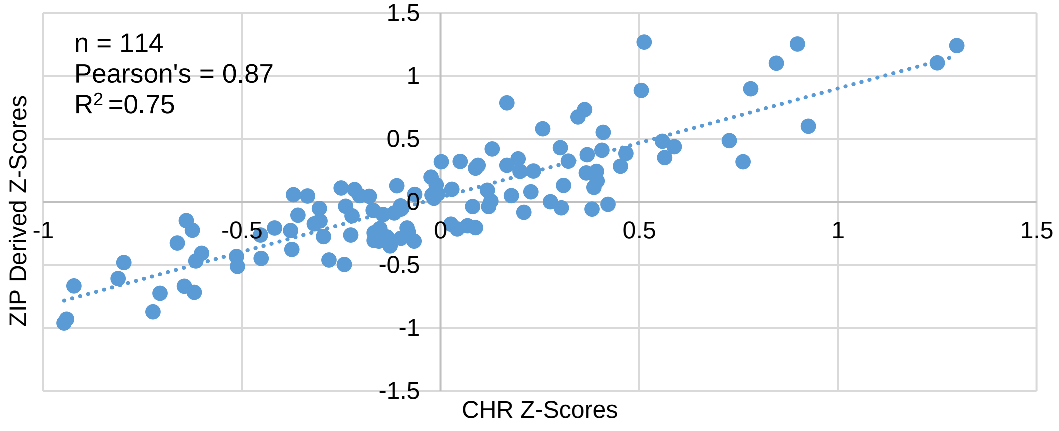

**Supplemental Digital Content Figure 3: Agreement Chart and Cross-Tabulation for 2015 CHR and ZIP Analog Derived Health Factors Quintiles for Missouri Counties**

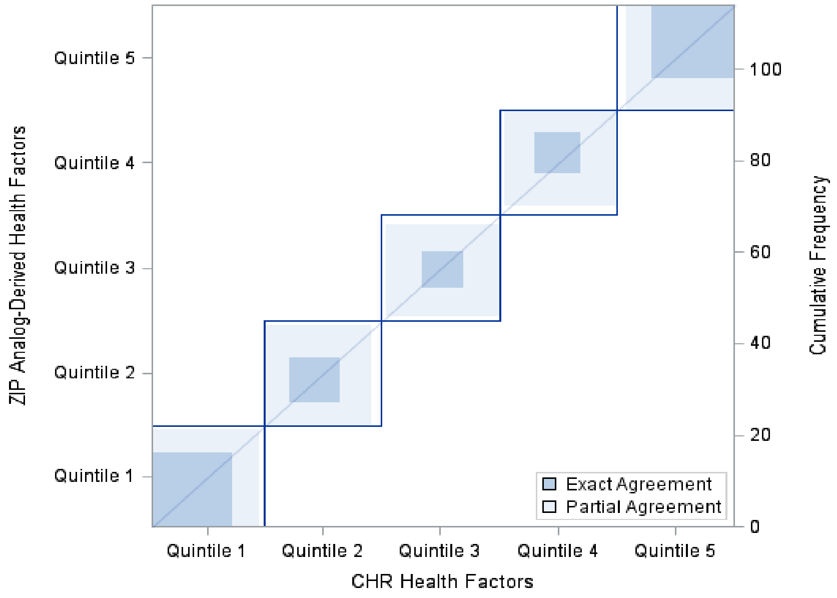


| | Q1 | Q2 | Q3 | Q4 | Q5 |
|---|---|---|---|---|---|
| **Q1** | 16 | 5 | 1 | 0 | 0 |
| **Q2** | 5 | 10 | 6 | 2 | 0 |
| **Q3** | 1 | 7 | 8 | 7 | 0 |
| **Q4** | 0 | 1 | 6 | 9 | 7 |
| **Q5** | 0 | 0 | 2 | 5 | 16 |

**Supplemental Digital Content Figure 4: Agreement Chart and Cross-Tabulation for 2015 CHR and ZIP Analog Derived Health Outcome Quintiles for Missouri Counties**

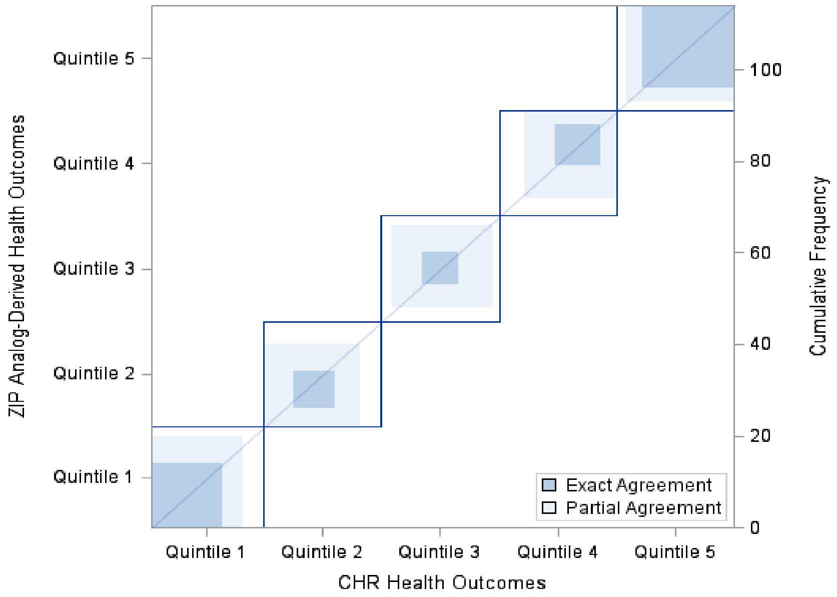


| | Q1 | Q2 | Q3 | Q4 | Q5 |
|---|---|---|---|---|---|
| **Q1** | 14 | 4 | 3 | 1 | 0 |
| **Q2** | 6 | 8 | 5 | 3 | 1 |
| **Q3** | 2 | 6 | 7 | 7 | 1 |
| **Q4** | 0 | 5 | 6 | 9 | 3 |
| **Q5** | 0 | 0 | 2 | 3 | 18 |

## Supplemental Digital Content Full Color Maps (Manuscript Figures 1-3)

Measuring sub-county differences in population health using hospital and census-derived data sets: the Missouri ZIP Health Rankings project

**Supplemental Digital Content Figure 5. County and ZIP-Level Rankings Derived from Hospital and Census-Derived Datasets vs. 2015 County Health Rankings Results**

**Health Factors**

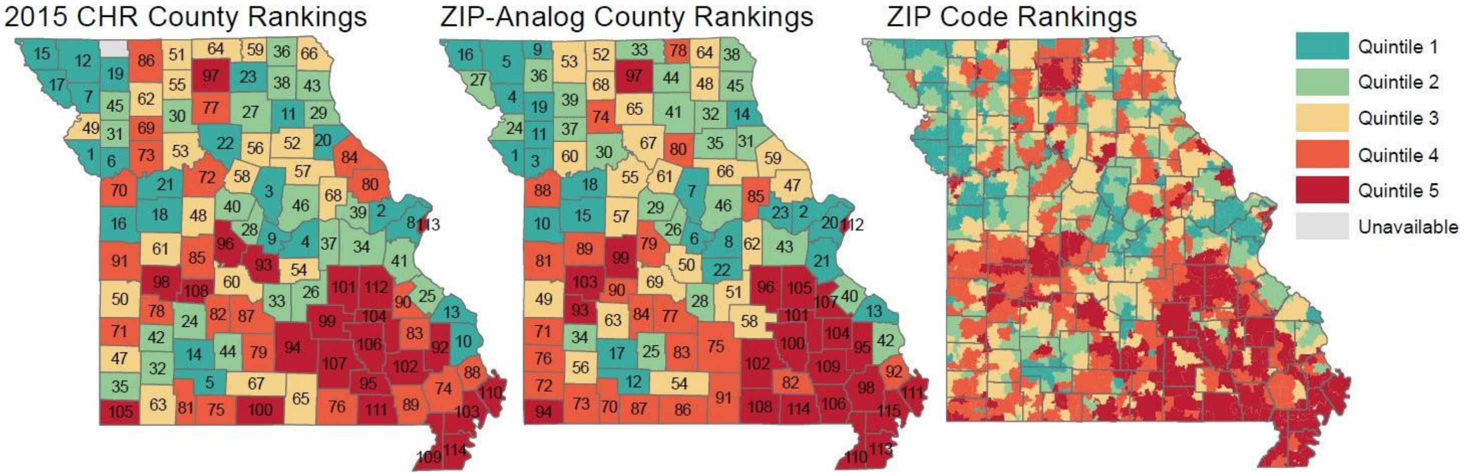


**Health Outcomes**

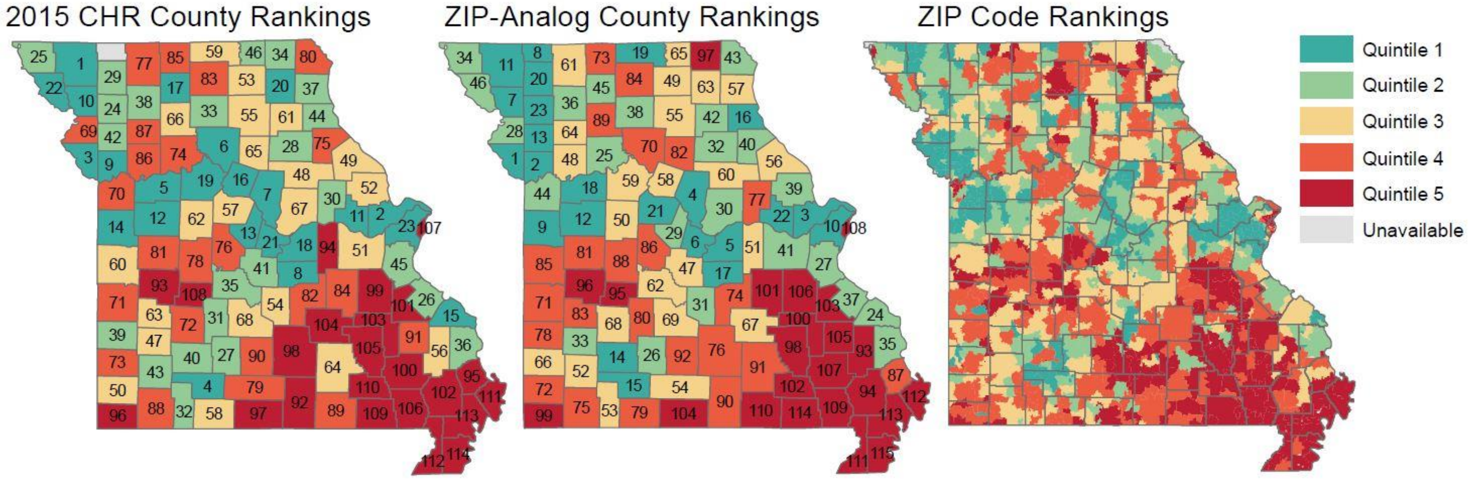

**Supplemental Digital Content Figure 6. Subcounty Variation in Health Factors and Outcomes in Urban St. Louis City & County, Missouri**

ZIP Ranking Range:
2 to 964 (of 976)

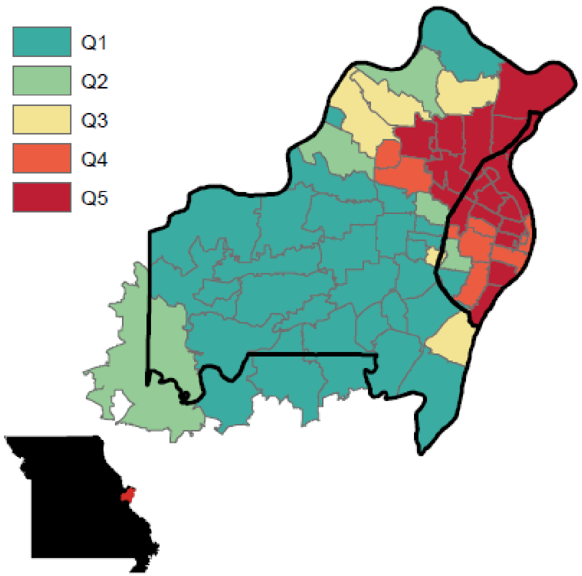


**St. Louis city and County Characteristics**

Health Factors Rank: **114 & 3 of 115**
Health Outcomes Rank: **111& 20 of 115**
2015 Combined Population **1,319,047**
Percent Non-White: **35.9**

**Top-Ranked ZIP Codes**

| ZIP | Name | Statewide Rank of 976 |
|---|---|---|
| 63005 | Chesterfield | 2 |
| 63038 | Glencoe | 4 |
| 63040 | Grover | 5 |

**Bottom-Ranked ZIP Codes**

| ZIP | Name | Statewide Rank of 976 | Top Health Determinant | Top Social Determinant |
|---|---|---|---|---|
| 63107 | North City E. | 964 | Asthma | Unemployment |
| 63113 | The Ville | 963 | Asthma | Unemployment |
| 63115 | North City W. | 962 | Asthma | Unemployment |
| 63133 | Pagedale-Wellston | 942 | Asthma | Single Parent Household |
| 63136 | Jennings | 929 | Asthma | Single Parent Household |
| 63134 | Berkeley | 914 | Asthma | Single Parent Household |

*Sources: Author's Calculations, 2016 County Health Rankings, and Roadmaps and U.S. Census Bureau QuickFacts*

**Supplemental Digital Content Figure 7. Subcounty Variation in Health Factors and Outcomes in Rural Franklin County, Missouri**

ZIP Ranking Range:
158 to 828 (of 976)

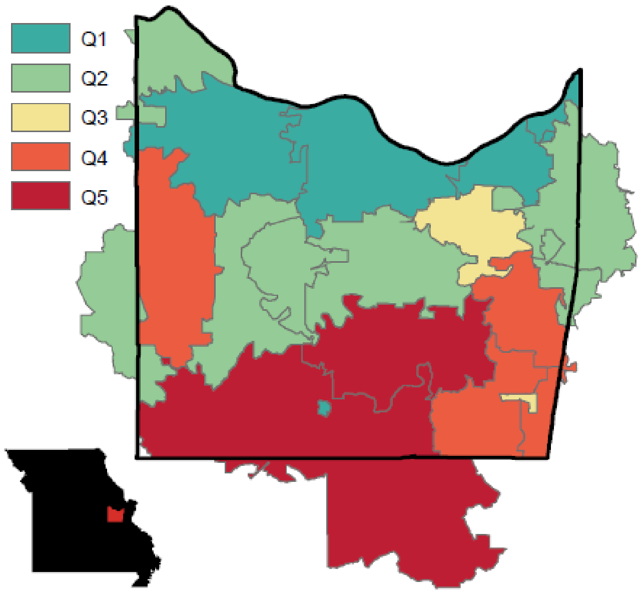


**Franklin County Characteristics**

Health Factors Rank: **38 of 115**
Health Outcomes Rank: **35 of 115**
2015 Population **102,426**
Percent Non-White: **3.4**

**Top-Ranked ZIP Codes**

| ZIP | Name | Statewide Rank of 976 |
|---|---|---|
| 63068 | New Haven | 158 |
| 63055 | Labadie | 165 |
| 63090 | Washington | 180 |

**Bottom-Ranked ZIP Codes**

| ZIP | Name | Statewide Rank of 976 | Top Health Determinant | Top Social Determinant |
|---|---|---|---|---|
| 63077 | Saint Clair | 828 | Smoking | Unemployment |
| 63080 | Sullivan | 810 | Mental Health | ED Visits |
| 63041 | Grubville | 753 | Premature Deaths | Childhood Poverty |
| 63072 | Robertsville | 686 | Smoking | Unemployment |
| 63037 | Gerald | 649 | Kidney Disease | Preventable ED Visits |
| 63060 | Lonedell | 645 | Smoking | Unemployment |

*Sources: Author's Calculations, 2016 County Health Rankings, and Roadmaps and U.S. Census Bureau QuickFacts*
